# Sodium valproate as a cause of recurrent transudative pleural effusion: a case report

**DOI:** 10.1186/1752-1947-3-51

**Published:** 2009-02-09

**Authors:** Stavros Tryfon, Maria Saroglou, Kosmas Kazanas, Charalambos Mermigkis, Kostas Psathakis, Nikolaos Galanis

**Affiliations:** 1 1st Pulmonary Clinic, G.H. "G. Papanikolaou", Thessaloniki, Greece; 2 General Army Hospital, Athens, Greece

## Abstract

**Introduction:**

There are few reported cases of neutrophilic pleural effusions associated with valproic acid therapy. Most of them are of eosinophilic exudates with or without blood eosinophilia.

**Case presentation:**

This case study describes a 70-year-old man with recurrent episodes of eosinophilic transudative pleural effusions associated with sodium valproate treatment. The recurrence of effusion after re-administration of the drug is strongly suggestive of an association between them. To the best of our knowledge, this is the first reported case with a pleural effusion with these characteristics caused by sodium valproate.

**Conclusion:**

This is the first report in the literature, with a full understanding of the etiology but with an unknown drug mechanism. This case report is of interest to different medical specialists (such as pulmonologists, neurologists, cardiologists) and pharmacologists.

## Introduction

This case study describes a 70-year-old man with recurrent episodes of neutrophilic transudative pleural effusions associated with sodium valproate re-administration. To the best of our knowledge, there are only five reported cases of pleural effusion associated with valproic acid therapy, but this is the first reported case of a pleural effusion with these characteristics.

## Case presentation

**Figure 1:**
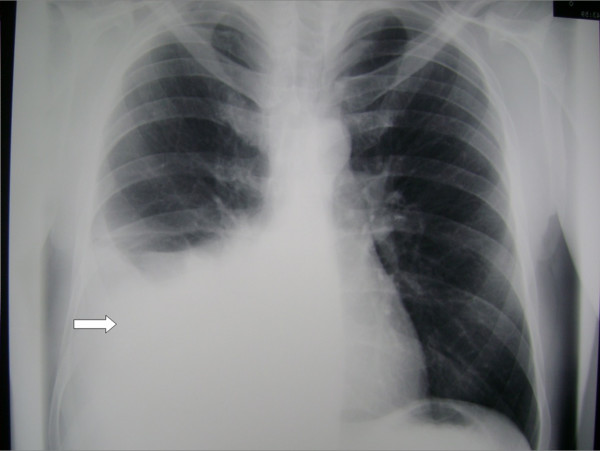
**Chest retro-anterior radiograph showing a large right-sided pleural effusion without lung parenchymal disorders**.

A 70-year-old male smoker (45 py), ex-farmer, was admitted to our department because of fever (38.8°C), dry cough and dyspnea. His symptoms commenced 5 days before his admission. He reported the same symptoms 8 months earlier when he had been admitted to another hospital. A chest radiograph had shown a large right-sided pleural effusion (Figure 1) and diagnostic thoracentesis had revealed a neutrophilic transudate. The fluid had been drained (700 ml of fluid) and the patient had left the hospital asymptomatic with a normal chest X-ray rejecting any further investigation.

His past history revealed atrial fibrillation (treated with digoxin), and post-traumatic epilepsy, after a road accident 1 year previously, treated since then with sodium valproate 500 mg/day.

On admission, the patient was febrile, tachypneic and looked moderately ill. Physical examination of the chest showed dullness on percussion at the middle and lower part of the right hemithorax as well as decreased breath sounds. The rest of the clinical examination was unremarkable. Standard laboratory studies demonstrated mild anemia (Ht = 32.2% and Hb = 9.7 mg/dl) and slightly increased erythrocyte sedimentation rate (ESR = 47 mm/h) and C-reactive protein (CRP = 2.8 mg/l). No leukocytosis or eosinophilia was observed. A postero-anterior chest X-ray showed a right-sided pleural effusion. Diagnostic thoracentesis revealed a neutrophilic transudative pleural effusion [total cell count = 100/μl, mainly neutrophils (65%)], glucose = 95 mg/dl, LDH = 30 IU/L, total proteins = 3 g/dl, albumin = 1 g/dl). A therapeutic thoracentesis (drainage of 1200 ml of fluid) resulted in dyspnea relief. Further laboratory investigation, including serum complement analysis, rheumatoid factor, antinuclear antibodies, thyroid hormones, antineutrophilic cytoplasmatic antibodies, immunoglobulin levels, serologic tests for hepatitis A, B and C, as well as for common viruses and atypical infectious agents, disclosed no apparent pathologies. HIV tests were also negative. Serum protein electrophoresis was also normal.

**Figure 2:**
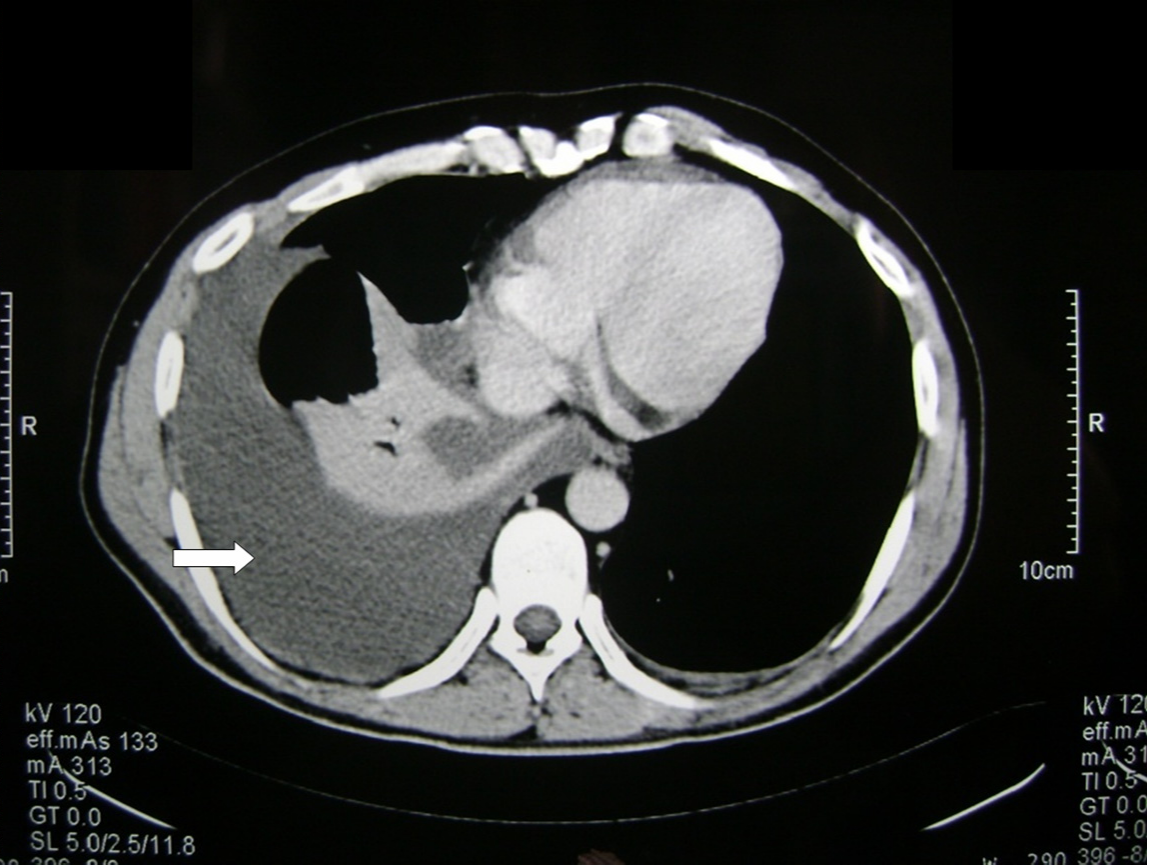
**Contrast-enhanced computed tomography of the chest revealing pleural fluid accumulation in both pleural cavities**. The occurrence of lung parenchyma is normal.

The echocardiography examination was normal. Contrast-enhanced computed tomography (CT) of the chest performed 1 day later revealed pleural fluid accumulation in both pleural cavities (Figure 2), while spiral CT pulmonary arterial angiography obtained simultaneously was negative for pulmonary embolism.

After exclusion of all possible reasons for the observed transudative pleural effusion, sodium valproate was discontinued and replaced with gabapentin (300 mg/day). During this period, the patient did not receive any other medication. Patient follow-up at 15 days, 1 and 2 months after his admission showed no relapse.

Seven months later, the patient had an epileptic episode and he changed his therapy on his own, from gabapentin to sodium valproate again. A month later, he was readmitted to our hospital because of dyspnea, fever, mild anemia with elevated CRP on laboratory tests and a right-sided pleural effusion on chest X-ray. Examination of the pleural fluid showed a transudative effusion with a small number of cells (90/μl), mainly neutrophils (87%).

Sodium valproate was discontinued and gabapentin was re-administered in higher doses (400 mg, twice a day), in order to avoid seizure relapse. This treatment was gradually followed by alleviation of the symptoms, elevation of hematocrit and normalization of CRP. No pleural fluid recurrence was observed after a sequential follow-up, up to 6 months later.

## Discussion

To the best of our knowledge, this is the first reported patient with a transudative pleural effusion due to valproic acid therapy. Adverse reactions to drugs produce only a small percentage of all pleural effusions; however, it is important to consider the possibility of drug-induced pleural disease after the exclusion of all other possible causes.

Valproic acid and its derivative – sodium valproate – are frequently used for the treatment of bipolar disorder, and are also used as an adjunct medication in patients with post-traumatic epilepsy and psychotic disorders such a schizophrenia [1]. Common side effects include nausea, weight gain, somnolence, and tremor. Hepatotoxicity has been reported in some cases. Serum eosinophilia is a possible but usually insignificant side effect.

A review of the literature revealed only five reported cases of pleural effusions associated with valproic acid therapy. Most of them were eosinophilic exudates with or without blood eosinophilia. The first case [2] referred to a patient with an exudative eosinophilic pleural effusion, which may have been caused by valproic acid or the concomitantly administered antipsychotic medication (chlorpromazine and fluphenazine) or by a potentiation effect. Since valproic acid and antipsychotics were used in combination, medication side effects [3] or interaction [4] should be considered as causative mechanisms. Two other case reports [5,6] described exudative eosinophilic pleural effusions with peripheral blood eosinophilia. No cause of the pleural effusion was found, but on cessation of valproic acid therapy, the pleural effusion and eosinophilia resolved in both patients. The other reported case [7] referred to a patient with a 'flu-like syndrome' and both pleural and pericardial (non-eosinophilic) effusion after long-term therapy with valproate. The last case was of lymphocytic pleural effusion [8].

Our patient presented with fever, fatigue, dyspnea and neutrophilic transudative pleural effusions in all three of his hospitalizations. No peripheral eosinophilia was observed. Valproic acid was suggested as the cause of the pleural effusion after exclusion of all other possible causes. Although the clinical picture of the patient, his laboratory blood tests as well as the presence of neutrophils in the pleural fluid suggested an inflammatory reaction, the rest of the characteristics of the pleural effusion were compatible with a transudate. A transudative effusion usually does not imply an inflammation, but instead disequilibrium of the hydrostatic and osmotic driving pressures between the pleural space and the capillary bed of the pleura. This was virtually excluded in our patient, since cardiac and renal functions as well as blood protein levels were all normal. A possible side effect of valproic acid on heart function was excluded as the echocardiography test was normal. Furthermore, it has been reported that intravenous injection of valproate at high concentrations, large doses and fast infusion rates produce no evidence of cardiotoxicity [9].

The explanation of the presence of a transudative pleural effusion in our patient is obscure. However, the most persuasive evidence that the whole clinical situation was due to the suspected drug was the recurrence of the pleural effusion whenever the regimen was administered and the disappearance of the effusion whenever the drug was discontinued. Indeed, after his final admission, the patient's clinical symptoms subsided with the discontinuation of valproic acid, while no pleural fluid recurrence was observed on follow-up thereafter.

The potential causative mechanism remains elusive, but, still, the recurrence of effusion after re-administration of the drug is strongly suggestive of an association. However, it has been suggested that viral infections (rhinopharyngitis) may induce these adverse events (fever, pleuritis) and additionally may cause clinically significant episodes of thrombocytopenia in these patients [10]. On the other hand, bone marrow suppression and pulmonary hemorrhage have only been reported [11] in valproate overdoses.

## Conclusion

This is the first reported case with a transudative pleural effusion due to valproic acid therapy, and this should be considered after all other possible causative factors have been excluded.

## Consent

Written informed consent was obtained from the patient for publication of this case report and any accompanying images. A copy of the written consent is available for review by the Editor-in-Chief of this journal.

## Competing interests

The authors declare that they have no competing interests.

## Authors' contributions

ST treated and followed up the patient. He took the informed concern from the patient for all diagnostic procedures, and wrote the first draft of the manuscript. ST, MS and KK performed the diagnostic procedures and analyzed and interpreted the patient data regarding the absence of cardiologic disease and the occurrence of adverse drug events. CM treated the patient at the second recurrence of pleuritis. CM and KP made the second part of the diagnostic procedures of the patient and have been involved in drafting the manuscript and then after revising it critically for important intellectual content. NG is the director of the clinic and organizes the methodology of diagnostic procedures, the treating algorithms and he has given final approval of the version to be published.

All authors read and approved the final revision of the manuscript.
